# A Novel Insight at Atherogenesis: The Role of Microbiome

**DOI:** 10.3389/fcell.2020.586189

**Published:** 2020-09-22

**Authors:** Tatiana V. Kirichenko, Yuliya V. Markina, Vasily N. Sukhorukov, Victoria A. Khotina, Wei-Kai Wu, Alexander N. Orekhov

**Affiliations:** ^1^Laboratory of Cellular and Molecular Pathology of Cardiovascular System, Research Institute of Human Morphology, Moscow, Russia; ^2^Laboratory of Angiopathology, Institute of General Pathology and Pathophysiology, Moscow, Russia; ^3^Department of Internal Medicine, National Taiwan University Hospital, Bei-Hu Branch, Taipei, Taiwan

**Keywords:** microbiome, atherosclerosis, inflammation, TMAO, mitochondria, lipoproteins

## Abstract

There is an important task of current medicine to identify mechanisms and new markers of subclinical atherosclerosis in order to develop early targets for the diagnosis and treatment of this disease, since it causes such widespread diseases as myocardial infarction, stroke, sudden death, and other common reasons of disability and mortality in developed countries. In recent years, studies of the human microbiome in different fields of medicine have become increasingly popular; there is evidence from numerous studies of the significant contribution of microbiome in different steps of atherogenesis. This review attempted to determine the current status of the databases PubMed and Scopus (until May, 2020) to highlight current ideas on the potential role of microbiome and its metabolites in atherosclerosis development, its mechanisms of action in lipids metabolism, endothelial dysfunction, inflammatory pathways, and mitochondrial dysfunction. Results of clinical studies elucidating the relationship of microbiome with subclinical atherosclerosis and cardiovascular disease considered in this article demonstrate strong association of microbiome composition and its metabolites with atherosclerosis and cardiovascular disease. Data on microbiome impact in atherogenesis open a wide perspective to develop new diagnostic and therapeutic approaches, but further comprehensive studies are necessary.

## Introduction

Today atherosclerosis remains one of the most important problems of current medicine, since it causes such widespread diseases as myocardial infarction, stroke, sudden death, and other common causes of disability and mortality in developed countries. The development of atherosclerosis has a long asymptomatic phase; the first clinical manifestations appear with a significant vascular lesion. Despite the fact that modern science understands the basic mechanisms of atherogenesis, individual factors, which determining the variability of the atherosclerosis progression, are still not well studied. In this regard, it is an important task to identify mechanisms and new markers of subclinical atherosclerosis in order to develop early targets for the diagnosis and treatment of this disease. Therefore, the need to search for additional factors of the pathogenesis of this disease remains relevant (Orekhov and Ivanova, 2017).

In recent years, studies of the human microbiome in different fields of medicine have become increasingly popular (Huttenhower et al., 2012). The relationship of the gut microbiome is shown with autoimmune, inflammatory (Belkaid and Hand, 2014; Opazo et al., 2018), neurodegenerative (Endres and Schäfer, 2018), infectious diseases (Lazar et al., 2018), and cancer (Villéger et al., 2019). Studies of microbiome to understand its relationship with cardiovascular diseases, in particular, mechanisms of atherogenesis has great potential (Kazemian et al., 2020).

The gut microbiome is the first largest and most complex community of microorganisms in the human body, which consists of bacteria, viruses, archaea, fungi, protozoa, and bacteriophages. It is an ecological community of microorganisms inhabiting the gastrointestinal tract along its entire length consists of a trillion bacteria and is encoded by more than 100 times more genes than the human genome (Mills et al., 2019). The gut microbiome is an important component of homeostasis involved in physiology and metabolism in human body (Sommer and Bäckhed, 2013). It participates in the digestion processes, providing fermentation of undigested substances, the synthesis of certain vitamins, energy metabolism, and regulation of the intestinal barrier function (Ahmad et al., 2019; Derovs et al., 2019). Recent studies have shown the key role of the microbiome in the functioning of the human immune system (Belkaid and Harrison, 2017). The composition of the intestinal microbiota depends on a number of factors, such as nutrition, lifestyle, gender, age, and the use of antibiotics (Jandhyala et al., 2015; Kundu et al., 2017).

This review reflects current ideas about the role of the microbiome in the processes of atherogenesis. Using the electronic databases PubMed and Scopus, a search was conducted for the keywords “microbiome,” “atherosclerosis,” “metabolites of the microbiome,” “TMAO” until May 2020. References’ lists of the identified reviews and original research articles were hand-selected for papers those may have been missed during primary search. Only articles published in English were selected. We found about 500 articles and focused on the studies on the influence of the microbiome on the pathogenesis of atherosclerosis.

## The Effect of Microbiome on Basic Pathways of Atherogenesis

**FIGURE 1:**
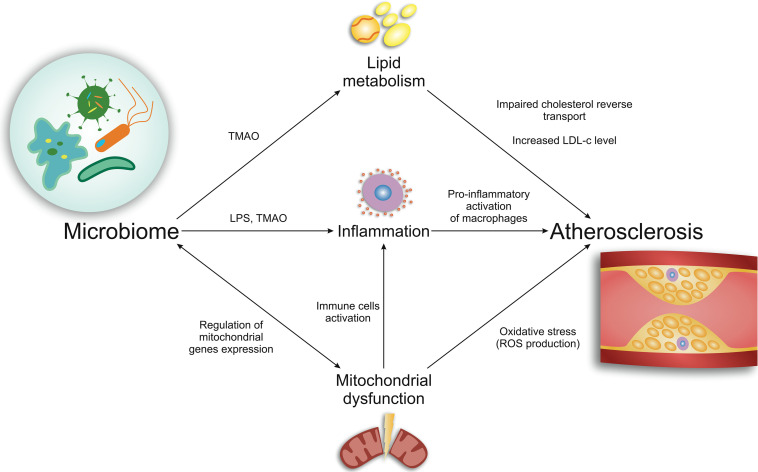
Brief scheme of microbiome effects in basic mechanisms of atherogenesis.

Numerous experimental and clinical studies investigate the role of microbiome and its metabolites in different steps of atherosclerosis development. Further in this article we will consider the main effects of the microbiome on lipid metabolism, inflammation, endothelial dysfunction, mitochondrial dysfunction during atherogenesis. Figure 1 represents a brief scheme of microbiome effects in basic mechanisms of atherogenesis.

### Microbiome and Lipid Metabolism

Lipid metabolism disturbance is the initial stage and one of the main reasons of atherosclerosis development, which is manifested in lipid accumulation in vessel wall (Pieczynska et al., 2020). The gut microbiota may disturb lipid metabolism both in mice and humans. Microbiome composition may further correlate with atherosclerotic lesion progression (Schoeler and Caesar, 2019). Intestinal microorganisms can modulate human metabolism at the level of small molecules, including the conversion of food components into hormone-like signals and biologically active metabolites. About 10% of small molecules circulating in the bloodstream have the origin of intestinal microbiome (Herrema et al., 2020). One of such biologically active metabolites of the intestinal microbiota, which may be involved in the development of atherosclerosis, is trimethylamine N-oxide (TMAO).

The increasing level of TMAO in plasma is correlated with the lipid deposition in blood vessels and endothelial dysfunction (Hardin et al., 2019). TMAO also may impair cholesterol reverse transport, which is promoted by bile acid biosynthesis (Sun et al., 2016). Bile acids can be ligands for nuclear farnesoid X receptor (FXR), which mediates the expression of genes regulating bile acid biosynthesis and excretion (Lefebvre et al., 2009). Members of cytochrome P450 superfamily, cholesterol 7 alpha-hydroxylase (CYP7A1) and cholesterol 27 alpha-hydroxylase (CYP27A1), are important bile acid synthetic enzymes responsible for maintaining cholesterol metabolism homeostasis (Sayin et al., 2013; Lau et al., 2017). Defective metabolism of bile acids and cholesterol is associated with atherosclerosis. Recently, the link of TMAO-induced atherosclerosis with bile acid metabolism was studied in apoE−/− mice on TMAO-containing diet (Ding et al., 2018). Specifically, it was elucidated that TMAO can repress hepatic bile acid synthesis pathway by activation of small heterodimer partner (SHP) and FXR, which can down-regulate of Cyp7a1 and Cyp27a1 gene expression (Koeth et al., 2013; Ding et al., 2018). Moreover, it was demonstrated that the levels of triglyceride, total cholesterol, low-density lipoprotein cholesterol (LDL-c), as well as, total plaque areas in the aortas in TMAO administration mice were significantly increased (Ding et al., 2018).

Human studies revealed the association of altered microbiota, represented mostly by some taxa from phylum Actinobacteria, with increased triglyceride level, decreased high-density lipoprotein cholesterol (HDL-c), and slightly changed LDL-c level. HDL mediates efflux of cholesterol from cells and then transports it to liver to be excreted from an organism (Fisher et al., 2012). It was elucidated in two studies on obese and healthy European individuals, that elevated fasting triglycerides and diminished HDL-c are associated with the low abundance of the gut microbiota (Cotillard et al., 2013; Nielsen et al., 2013). Furthermore, as a result of the systemic analysis of blood lipid levels, body mass index, and gut microbiota, conducted in 893 participants from the LifeLines-DEEP cohort, it was suggested that the gut microbiota may influence only certain aspects of lipid metabolism and distinct classes of lipoproteins, such as HDL and very low-density lipoproteins (VLDL) (Fu et al., 2015). Enrichment in Bacteroides, Eubacterium, and Roseburia microbes, which are common microbiota in healthy people, can correlate with decrement of body mass index and plasma triglycerides and increment of plasma HDL (Nakaya and Ikewaki, 2018). In addition, the full suppression of gut microbiota may enhance macrophage-to-feces reverse cholesterol transport (Canyelles et al., 2018). However, there are a lot of blind spots in the TMA/FMO3/TMAO/bile acids axes in human. Thus, further studies that help to clarify the exact role of gut microbiome and TMAO in cholesterol and bile acid metabolism in human, are necessary.

### Microbiome and Inflammation

Atherosclerosis is a chronic inflammatory disease, which is characterized by the accumulation of monocytes and lymphocytes within the arterial wall, the release of chemokines, interleukins, and activation of pro-inflammatory pathways within endothelial cells (Libby, 2012; Tuttolomondo et al., 2012; Nikiforov et al., 2019). Possible association of gut microbiome composition, diet, atherosclerosis, and inflammation is under thorough investigation (van den Munckhof et al., 2018). For example, saturated fat rich diet can dramatically change the gut microbiome composition, elevating the intestinal absorption of gut-derived microbial products that lead to an increased concentration of plasma lipopolysaccharide (LPS), which is called metabolic endotoxemia (Sonnenburg and Bäckhed, 2016). The activation of inflammatory signaling pathways in the intestine is one of the factors determining the influence of the gut microbiome on the development of atherosclerosis. LPS, the main component of the outer membrane of gram-negative bacteria, can penetrate the bloodstream as a result of the intestinal mucosa barrier function disturbance (Ahmad et al., 2019). Consequently, metabolic endotoxemia provokes macrophage activation via TLR4/MyD88/TRIF signaling (Caesar et al., 2015). TLR4/MyD88/TRIF is activated by the lipid A component of LPS and this causes inflammatory signaling pathways (Herrema et al., 2020; Caesar et al., 2015). Thus, microbiome alteration based on a diet type may influence on pro-inflammatory activation, which is correlated with atherosclerosis since pro-inflammatory macrophages are one of the key cells in atherogenesis. The role of microbiome composition in pro-inflammatory status was further demonstrated in mice on high-fat diet, which is positively correlated with metabolic endotoxemia and endothelial dysfunction (Toral et al., 2014). It was shown, that *Lactobacillus coryniformis* administration as a probiotic promoted the increase in the intestinal mucus glycoprotein mucin-3, which restores gut barrier function and subsequently decreases plasma LPS level. Moreover, *L. coryniformis* administration lowered IL-6 and IL-1β expression in aorta of these mice. In addition, it was elucidated in germ free mice that the absence of microbiota has beneficial effect on arterial lesion and helps to decrease inflammatory cytokines in macrophages (Kasahara et al., 2017).

### Microbiome and Mitochondrial Dysfunction

Genetic predisposition to atherosclerosis development is widely studied nowadays. Mitochondrial dysfunction is one of the possible genetic factors that may lead to the progression of atherosclerosis (Sazonova et al., 2017; Orekhov et al., 2020). In previous studies the association of mitochondrial heteroplasmy and atherosclerosis was demonstrated. Several variants of mitochondrial heteroplasmy were found in atherosclerotic lesions of human aorta (Sazonova et al., 2015; Sobenin et al., 2019) as well as the correlation of these mitochondrial mutations in white blood cells with carotid atherosclerosis was demonstrated in some clinical trials (Kirichenko et al., 2018, 2020a). Inflammatory changes leading to the progression of atherosclerosis, the manifestation of CVD and the development of their complications, develop with age due to many factors, including oxidative stress caused by mitochondrial dysfunction as well as inflammatory processes caused by changes in the composition of the microbiota described above (Ostan et al., 2016; Ferrucci and Fabbri, 2018). At the same time, association of mitochondrial dysfunction and alterations of gut microbiota are studied in several trials (Ma et al., 2014; Hirose et al., 2017). It was demonstrated in subjects with Crohn’s disease that downregulation of mitochondrial proteins, which indicates the central role of mitochondria dysfunction in the pathogenesis of inflammatory bowel disease, is associated with a depletion of butyrate producers, suggesting a signaling role for butyrate in host mitochondrial genes expression (Mottawea et al., 2016). It is assumed that gut microbiota signaling to mitochondria leads to the activation of immune cells and inflammation, and changes the function of the epithelial barrier (Jackson and Theiss, 2019). It was proposed that production of toxins by dysbiotic gut microbiome activates neuronal innate immunity and inflammatory processes that lead to the development of neurodegenerative processes in patients with Parkinson’s disease (Cardoso and Empadinhas, 2018). As for the possible relationship of the microbiome and mitochondria in the pathogenesis of atherosclerosis, the mechanisms of their interaction are not well studied at present. However, we can assume the combined influence of these factors on the development of atherosclerosis, since mitochondria are bacterial in origin, and therefore microbiome products can affect their functions, stimulating processes that play an important role in atherogenesis (Clark and Mach, 2017; Bajpai et al., 2018). In particular, gut microbiome influences the production of reactive oxygen species (ROS) in mitochondria and regulates their inflammatory activity (Saint-Georges-Chaumet and Edeas, 2016; Clark and Mach, 2017). Overproduction of ROS stimulates inflammatory reactions which affect the development of atherosclerosis (Forrester et al., 2018). On the other hand, in mice, engineered for overproduction of ROS in mitochondria and in aged mice, the composition of the intestinal microbiota changed compared to the initial one, Shannon diversity significantly decreased, that demonstrates the reverse effect of mitochondrial dysfunction on microbiome. At the same study, in mice treated with N-acetylcysteine or engineered to produce more mitochondrial catalase, ROS production decreased significantly that led to significant increase in Shannon diversity (Yardeni et al., 2019). So, the microbiome-mitochondria interaction may be an important factor in mechanisms of atherogenesis, but further studies are required.

## The Role of Microbiome Metabolites in the Development of Atherosclerosis

### TMAO Metabolism

The prevailing phyla of bacteria compounded the gut microbiome are the Actinobacteria, Bacteroides, Firmicutes, Proteobacteria, and Verrucomicrobiota. The balance between Bacteroides and Firmicutes may determine the health of gastrointestinal tract (Jama et al., 2019). Shift to Firmicutes domination may lead to dysbiotic environments, which is beneficial to TMAO accumulation. TMAO is produced in liver by flavin-containing monooxygenase 3 (FMO3) from the precursor trimethylamine (TMA), which is generated in gut from metabolic intermediates incorporating the TMA-moiety, such as betaine, γ-butyrobetaine, choline, phosphatidylcholine, and L-carnitine (Chen K. et al., 2017). TMA lyases are gut microbe enzymes, which convert choline to TMA. An inhibition of these lyases may reduce atherosclerosis in mice (Canyelles et al., 2018).

Circulating plasma TMAO levels are determined by several factors, including the consumption of its metabolic precursors, medication, and liver flavin monooxygenase activity (Janeiro et al., 2018). The level of TMAO largely depends on the composition of the intestinal microbiome. The predominant bacterial types are Bacteroides and Firmicutes, and Actinobacteria, Proteobacteria, and Verrucomicrobia are less common. The splitting processes of the main source of TMAO, choline, are regulated by the intestinal microbiota, in particular, the phylum Firmicutes and Proteobacteria, and the genera *Anaerococcus hydrogenalis*, *Clostridium asparagiforme*, *Clostridium hathewayi*, *Clostridium sporogenes*, *Escherichia fergusonii*, *Proteus penneri*, *Providencia rettgeri*, and *Edwardsiella tarda*. The main microbial species responsible for the degradation of another TMAO source of L-carnitine are the phylum Proteobacteria and Bacteroides and the family Prevotellaceae (Zhu et al., 2020).

Recent clinical studies confirm the close relationship of atherosclerosis with the level of TMAO. Increased plasma TMAO levels in patients are associated with an increased risk of adverse cardiovascular events such as stroke, heart attack, and death (Tang et al., 2013). It was found in the study on patients with acute coronary syndrome (ACS) that elevated TMAO value is a high predictor of long-term mortality risk (Senthong et al., 2016b).

### TMAO and Foam Cells

The early stage of atherosclerosis is the accumulation of foam cells in the intima of the arteries. Most foam cells come from macrophages that can regulate lipoprotein metabolism (Poznyak et al., 2020). The association of TMAO with stimulation of atherosclerosis progression was first demonstrated in animal models where TMAO led to the formation of foam cells in atherosclerotic lesions by increasing expression of the CD36 and SR-A1 scavenger receptors (Wang et al., 2011). It is known that the main reason for the formation of foam cells is the excessive influx of modified LDL and the accumulation of cholesterol esters in macrophages. Macrophages express various CD36 and SR-A1 scavenger receptors, as well as the lectin-like receptor oxLDL-1 (LOX-1), with affinity to oxidized low-density lipoproteins (oxLDL). This in turn induces the formation of foam cells (Chistiakov et al., 2017). It has been shown that macrophage foam cell formation was increased in apoE−/− mice on TMAO-, choline-, or L-carnitine-fed diets (Wang et al., 2011). Up-regulation of macrophage scavenger receptors CD36 and SR-A by TMAO is one the plausible mechanisms underlying this process (Koeth et al., 2013). TMAO is probably involved in the process of atherogenesis, contributing to the migration of macrophages, the accumulation of ox-LDL in them, and their transformation into foam cells (Mohammadi et al., 2016; Janeiro et al., 2018). In addition, studies revealed that TMAO positively regulates the expression of VCAM-1 in endothelial cells, leading to an increase in monocyte adhesion, which is an early sign of foamy cell formation (Ma et al., 2017).

### Effect of TMAO on Inflammation and Endothelial Dysfunction

Trimethylamine N-oxide is believed to enhance atherosclerosis by influencing inflammatory processes in vascular wall by activating the inflammatory pathway (Ahmad et al., 2019). The processes of inflammation are involved in all stages of the development of atherosclerosis, from the initial lesions to the terminal stage of thrombotic complications. Most often, the processes begin with inflammatory changes in the vascular endothelium, characterized by the expression of VCAM-1 and monocyte adhesion (Ma et al., 2017). Elevating plasma level of TMAO is associated with the high risk of atherosclerosis and cardiovascular disease due to chronic inflammation and the recruitment of leukocytes to endothelium (Al-Obaide et al., 2017). TMAO promotes macrophage migration and an increase in the expression of inflammatory cytokines such as TNF-α and IL-6 and a decrease of the expression of the anti-inflammatory cytokine IL-10 (Zhu et al., 2020). In response to inflammatory activation, macrophages can penetrate the vascular endothelium and accumulate in the intima, thereby causing the formation of plaques (Chistiakov et al., 2017). It was shown that an increase in TMAO levels led to the activation of a mitogen-activated protein kinase (MAPK), a signaling cascade of nuclear factor-κB (NF-κB), and also to over-expression of pro-inflammatory genes, inflammatory cytokines, adhesion molecules, and chemokines, so TMAO elevation stimulates the inflammatory response within endothelial and smooth muscle cells (Seldin et al., 2016). In addition, TMAO can trigger vascular inflammation by the NLRP3 mechanism – inflammasome is an IL-1β family cytokine-activating protein complex, consisting of the pattern recognition receptor NLRP3, adaptor protein apoptotic speck-like protein, and inactive pro-caspase-1, and involved in the regulation of inflammatory response (Chen M. L. et al., 2017). It was also shown, that TMAO can activate inflammation via NOD-like receptor family and may lead to NLRP3 inflammasome activation through the SIRT3-SOD2-mtROS signaling pathway in human umbilical vein endothelial cell and aortas from apoE−/− mice. It was demonstrated at the same study that TMAO may induce the expression of cyclooxygenase-2 (COX-2), IL-6, E-selectin, and intercellular adhesion molecule-1 (ICAM-1) in primary human aortic endothelium cells. It can also promote production of COX-2, IL-6, TNF-α, and ICAM-1 in vascular smooth muscle cells (Matsumoto et al., 2020).

In addition to the activation of NLRP3 – inflammasome, TMAO can affect the processes leading to endothelial dysfunction, which is also an important factor in the development of atherosclerosis. In experiments on cell cultures, it has been shown that TMAO can induce oxidative stress and activate NLRP3 – inflammasome, results in the release of inflammatory cytokines, and reduce the level of endothelial nitric oxide synthase (eNOS) and the production of nitric oxide (NO) (Sun et al., 2016). Intracellular inflammation and oxidative stress reactions lead to the formation of ROS and a decrease in NO. In addition, this leads to inhibition of the function of circulating endothelial progenitor cells (EPCs), which also has a negative effect on maintaining the function of the vascular wall. All these pathophysiological processes ultimately lead to endothelial dysfunction and the development of atherosclerosis (Chou et al., 2019).

### TMAO in the Processes of Thrombosis

The effect of TMAO on platelet activation processes and following thrombus formation, which play an important role in atherosclerotic events, has been shown in several studies (Kasahara and Rey, 2019). A dose-dependent effect was revealed between the increased content of TMAO in blood plasma and platelet hyperreactivity. Most likely, this is due to the influence of TMAO on the release of intracellular Ca^2+^ stores from platelets and their activation as a result of stimulation of ADP, thrombin, collagen, arachidonic acid (Zhu et al., 2016).

### Other Microbiome Metabolites

Besides TMAO, which makes a significant contribution to the development of atherosclerosis, there are other biologically active metabolites of the gut microbiota. One of them is short chain fatty acids (SCFA), such as acetate, butyrate and propionate, resulting from the fermentation of undigested dietary fiber. They play an important regulatory role, act as substrates in cholesterol and lipid metabolism (Chen et al., 2020; Zeng and Tan, 2020). SCFA have anti-inflammatory effects by inhibiting the migration and proliferation of cells of the immune system and the production of cytokines, which can weaken the progression of atherosclerosis (Ohira et al., 2017).

Other metabolites of the gut microbiome are aromatic amino acids. The metabolism of phenylalanine, tyrosine, tryptophan, and histidine leads to the formation of compounds that can also affect the development of cardiovascular diseases. For example, tryptophan metabolism results in indole ethanol (IE), indolepropionic acid (IPA) and indole acrylic acid (IA), which are absorbed through the intestines and have anti-inflammatory activity, which can be a beneficial for CVD development (Kasahara and Rey, 2019). Other uremic toxins, indoxyl sulfate and p-cresyl sulfate, are metabolites derived from tyrosine, phenylalanine, and tryptophan by gut microbiota (Nallu et al., 2017; Pereira-Fantini et al., 2017). Indoxyl sulfate is produced in liver from the *Escherichia coli* metabolite indole. Indoxyl sulfate can activate IL-6 expression in both endothelium and smooth muscle cells via organic anion transporters/aryl hydrocarbon receptor/NF-κB pathway that has been elucidated in mice studies (Matsumoto et al., 2020). It has also been studied that indoxyl sulfate can increase ICAM-1, TNFα, and monocyte chemoattractant protein-1 (MCP-1) expression (Tumur et al., 2010). Elevated plasma p-cresyl sulfate is a risk factor of cardiovascular disease in patients with chronic kidney disease, since it can up-regulate MCP-1, ICAM-1, TNF-α, and vascular cell adhesion molecule-1 expression in endothelium cells. It was also demonstrated that increased p-cresyl sulfate level may promote atherosclerotic lesion in apoE−/− mice with chronic kidney disease (Jing et al., 2016). Furthermore, it was shown in human study, that oat and barley beta-glucans, prebiotic fibers with proven cholesterol-lowering activity, significantly reduced LDL and total cholesterol, and serum p-cresyl sulfate levels, which may have positive effect on endothelial function (Cosola et al., 2017).

### Circadian Rhythms of Microbiome and Atherogenesis

It is believed that circadian rhythms affect the development of atherosclerosis since immune cell activity depends on the circadian clock (Winter and Soehnlein, 2018). Conventional cardiovascular risk factors such as blood pressure, pulse rate, endothelial function are also subjected to diurnal variation (Kawano et al., 2002; Paschos and FitzGerald, 2010). The composition of gut microbiota, which is associated with atherogenesis, is also characterized by diurnal variations. However, it is still not entirely clear how the circadian clock and gut microbiota, in particular bacterial metabolites such as TMAO, are associated with the progression of atherosclerosis. Clock (Circadian Locomotor Output Cycles Kaput) and Bmal1 [Brain and Muscle ARNT (aryl hydrocarbon receptor nuclear translocator) -Like 1], two major circadian clock genes, play important regulatory role in atherogenesis, expression of Bmal1 and Clock decreased in patients with atherosclerosis (Winter and Soehnlein, 2018; Man et al., 2020). TMAO induces increased expression of lncRNA-NEAT1, Clock and Bmal1 and inhibits the MAPK pathways (Wu et al., 2019a). Some researchers suggest that pro-atherosclerotic changes in the composition of the microbiota are due to the influence of toxic products, in particular, the environmental pollutant acrolein. Acrolein exposure increased MMP9, decreased Clock and Bmal1, and activated MAPK-pathways in human umbilical vein endothelial cells in plasma of apolipoprotein-E deficient mice fed a high fat diet with acrolein. At the same, in this model feeding with acrolein changed the composition of the gut microbiota – increase of Firmicutes and decrease of Bacteroidetes and these changes correlated with atherosclerotic plaque, MMP9 and Bmal1 levels (Wu et al., 2019b). Some analogy in the change in daily fluctuations in the composition of the intestinal microbiota was found in the study on a mouse model of diabetes. Rhythmic oscillations of gut microbiota were observed in diabetic mice as well as analysis of circulating metabolites showed changes in the daily concentration of metabolites of the histidine pathway and the metabolism of betaine, methionine, cysteine, followed by an increase in TMAO production (Beli et al., 2019). Moreover, in the study of atherosclerosis, associated with infection of *P*orphyromonas *gingivalis* that causes periodontitis, circadian clock disruption enhanced atherosclerosis progression in Bmal1−/− ApoE−/− mice (Xie et al., 2020). Despite of a number of studies on the influence of circadian rhythms on atherogenesis and the composition of gut microbiome and its metabolites, the mechanism of the influence of diurnal variations in the microbiome on the development of atherosclerosis is still an actual topic for research in the near future.

## The Association of Human Microbiome With Atherosclerosis and CVD

It is known that one of the surrogate markers and an indicator of early atherosclerosis, widely used in epidemiological and interventional studies, is the carotid intima-media thickness (cIMT) measured in common carotid arteries by high-resolution ultrasonography (Baldassarre et al., 2010; Kirichenko et al., 2020b). On the other hand, there is increasing evidence showing the association of TMAO with an increased risk of cardiovascular disease development (Senthong et al., 2016a; Haghikia et al., 2018). Table 1 summarizes the clinical studies on the association of TMAO with atherosclerosis and CVD.

**TABLE 1:** Clinical evidences on association of TMAO with atherosclerosis and CVD.

Study design	Results	References
**TMAO and cIMT**		
CARDIA study: 817 patients aged 33–45 years	No significant correlation of TMAO level with cIMT	Meyer et al., 2016
TULIP study: 220 participants with BMI > 27 kg/m^2^ or previous diagnosis of impaired glucose tolerance; 9-months lifestyle improvement (diet and sports)	TMAO level correlated with cIMT at baseline (*p* < 0.0001) and was associated with cIMT increase (*p* = 0.03) as well as age (*p* < 0.0001) and visceral fat mass (*p* = 0.0001)	Randrianarisoa et al., 2016
175 CVD-free HIV-infected patients	No significant correlation TMAO and cIMT; Trend toward higher TMAO level in patients with cIMT > 0.9 mm (*p* = 0.087)	Montrucchio et al., 2020
**TMAO in patients with CAD**		
353 patients with evidence of significant coronary artery disease defined by diameter stenosis ≥50% in vessels ≥1.5 mm	TMAO level correlated with coronary atherosclerosis measured by angiography using SYNTAX score (*p* < 0.0001)	Senthong et al., 2016b
4007 participants mean aged 63 years old at least single-vessel CAD, 3 years of follow-up	TMAO level was a strong predictor of increased risk of myocardial infarction, stroke and death (*p* < 0.001)	Tang et al., 2013
1612 patients in the Swiss ACS Cohort	TMAO levels were associated with a graded increase (log rank *p* < 0.001) in risk of major adverse cardiac events	Li et al., 2017
1-year prospective trial of 608 patients from the “Prospective Cohort with Incident Stroke” study (PROSCIS) with first-ever stroke	TMAO level >4.86 μM was associated with an increased cardiovascular risk in patients aged over 66 years (*p* < 0.01)	Haghikia et al., 2018

The positive correlation of cIMT values with circulating TMAO levels, regardless of gender, age, and visceral fat mass was demonstrated in 220 participants of Tübingen Lifestyle Intervention Program. In this study, lifestyle intervention led to improvement of cardiovascular risk factor, but TMAO level did not change significantly, at the same time, cIMT decreased significantly only in participants with large TMAO decrease (Randrianarisoa et al., 2016). It was shown in another study in 175 CVD-free HIV-infected patients, who have a higher cardiovascular risk than non-infected individuals due to HIV infection, that cIMT was higher toward higher TMAO concentration (Montrucchio et al., 2020). There are some contradictions regarding the role of TMAO in early atherosclerosis, probably caused by insufficient data. In some studies, patients with atherosclerosis did not have an elevated serum TMAO level compared with healthy participants, but an increase in the content of its precursor, L-carnitine, was found (Skagen et al., 2016). Another clinical study in 817 patients aged 33–45 years showed that the concentration of TMAO was associated neither with cIMT nor with coronary calcium measured by computed tomography, which may indicate a slight effect of TMAO in the development of early atherosclerosis in comparison with other studies in older people (Meyer et al., 2016). In addition to studies on the association of the TMAO level and cIMT in patients with subclinical atherosclerosis, this indicator is also used for cardiovascular risk prognosis in patients with clinical manifestations of atherosclerosis, namely, coronary artery disease (CAD). In particular, it was shown in over 4000 participants mean aged 63 years old at least single-vessel CAD, that elevated plasma concentration of TMAO was a strong predictor of increased risk of adverse cardiovascular events such as myocardial infarction, stroke and death after 3 years of follow-up. At baseline, the addition of TMAO to traditional risk factors as a covariate significantly improved cardiovascular risk estimation (Tang et al., 2013). These data may indicate the effect of TMAO at later stages of the atherosclerosis development, in high-risk subgroups, as well as among older people.

In addition to TMAO, the association of microbiome composition and other microbiome characteristics with atherosclerosis and CVD are also being investigated in epidemiological studies. For example, sequencing of gut microbiome in 218 individuals with atherosclerosis-based cardiovascular disease and 187 healthy controls demonstrates that among major genera of the gut microbiome, there is a relative reduction in Bacteroides and Prevotella, and enrichment in Streptococcus and Escherichia in atherosclerotic patients. At the same time, the abundance of Enterobacteriaceae including *E. coli*, *Klebsiella* spp., *Enterobacter aerogenes*, *Eggerthella lenta*, and *Ruminococcus gnavus* was higher in atherosclerotic patients than in control samples (*q*-value < 0.05 for all) (Jie et al., 2017). The study in patients, who undergone surgery to excise an atherosclerotic plaque, in comparison with CVD-free participants, shows, that genus Collinsella was enriched in patients with symptomatic atherosclerosis and Eubacterium and Roseburia were enriched in controls (*p* < 0.05) (Karlsson et al., 2012). In another study, the composition of gut microbiota was compared in 39 patients with CAD, 30 patients with coronary risk factors and 50 healthy individuals; the order of Lactobacillales was increased (*p* < 0.001) and the phylum Bacteroidetes (Bacteroides + Prevotella) was decreased (*p* < 0.001) in CAD group (Emoto et al., 2016).

Besides gut microbiome, oral cavity is also an important source of bacteria which affect atherosclerosis and CVD development. So, it was demonstrated in almost 12,000 study participants that poor dental hygiene increases risk of cardiovascular disease events and cardiovascular disease death (*p* = 0.001, for trend) (de Oliveira et al., 2010). INVEST study demonstrates the association of periodontal bacterial burden with cIMT in 657 patients over 55 years old without history of stroke, myocardial infarction, or chronic inflammatory diseases. cIMT increased across tertiles of etiologic bacterial burden which summing the standardized values for the bacteria causative of periodontal disease (*P. gingivalis, Tannerella forsythensis, Aggregatibacter Actinomycetemcomitans*, and *Treponema denticola*) in the subgingival plaque (*p* = 0.03) as well as across predominance of causative bacteria over other health-associated bacteria (*p* = 0.002) (Desvarieux et al., 2005). It was also demonstrated in the study of oral microbiota in atherosclerotic patients that abundance of Anaeroglobus was significantly higher in patients with symptomatic atherosclerosis than in control group (Fak et al., 2015). Additionally, it was detected in the study in 492 participants, that median salivary levels of *A. actinomycetemcomitans* were significantly higher in a group of patients with stable coronary artery disease (*p* = 0.014) and in a group with ACS (*p* = 0.044) than in control group (Hyvärinen et al., 2012).

## Conclusion

This review highlighted currently published data on the impact of microbiome in basic steps of the pathogenesis of atherosclerosis. Studies investigating the association of human microbiome with subclinical atherosclerosis and cardiovascular disease demonstrate prognostic value of TMAO in cardiovascular risk assessment and revealed several types of bacteria affecting the development of atherosclerosis and its clinical complications. Data on microbiome impact in atherosclerosis development open a wide perspective to develop new diagnostic and therapeutic approaches which can be especially relevant in case of subclinical atherosclerosis. Further comprehensive study of the mechanisms of microbiome impact in atherosclerosis development is still necessary.

## Author Contributions

AO and W-KW: conceptualization and design the review, and review final version approval. TK, VS, YM, and VK: bibliographic research. TK, VS, and YM: writing – original draft preparation. TK, VS, and VK: table and figure design. W-KW and TK: supervision. AO: funding acquisition. All authors contributed to the article and approved the submitted version.

## Conflict of Interest

The authors declare that the research was conducted in the absence of any commercial or financial relationships that could be construed as a potential conflict of interest.
